# Exogenous gonadotropin-releasing hormone counteracts the adverse effect of scrotal insulation on testicular functions in bucks

**DOI:** 10.1038/s41598-022-11884-4

**Published:** 2022-05-12

**Authors:** Mohamed S. Yousef, Gaber A. Megahed, Gamal F. Abozed, Mohamed Hayder, Hanan H. Abd-Elhafeez, Mohamed S. Rawy

**Affiliations:** 1 Department of Theriogenology, Faculty of Veterinary Medicine, Assiut University, Assiut, Egypt; 2 Animal Production Research Institute, Cairo, Egypt; 3 Department of cell and tissues, Faculty of Veterinary Medicine, Assiut University, Assiut, Egypt; 4 Department of Theriogenology, Faculty of Veterinary Medicine, Minia University, Minia, Egypt

**Keywords:** Zoology, Reproductive biology

## Abstract

This study determined the effects of scrotal insulation on testicular functions in bucks and evaluated the impact of exogenous gonadotropin-releasing hormone (GnRH) administration before scrotal insulation on sperm production and testicular vascular dynamics. Twelve bucks were randomly divided into three groups: scrotal-insulated animals without GnRH treatment (INS), scrotal-insulated animals treated previously with GnRH (GnRH + INS), and animals without insulation as controls (CON). Doppler ultrasonography was used to evaluate testicular vascular changes, and semen samples were collected to assess seminal parameters. Testicular samples were collected from slaughtered bucks at the end of the experiment for histological investigations and immunohistochemical analysis for caspase 3 (apoptotic marker), and a vascular endothelial growth factor (VEGF; hypoxic marker) evaluation. Sperm motility drastically decreased (33%) in the INS group on day 8 compared with those in the GnRH + INS and CON groups (58% and 85%, respectively). Testicular blood flow significantly decreased for 3 and 2 weeks in the INS and GnRH + INS groups, respectively. The pulsatility index (PI) reached pretreatment values at 5 and 4 weeks after insulation in the INS and GnRH + INS groups, respectively. The resistance index (RI) values increased in both insulated groups for the first 2 weeks and decreased to control values 4 weeks after insulation. However, the maximum velocity (VP) started to increase reaching pretreatment values by the 5th and 3rd weeks after insulation in the INS and GnRH + INS groups, respectively. Histological investigations showed a marked reduction in lipid inclusions in Sertoli cells in the GnRH + INS group compared with those in the INS group. The distributions of both caspase 3 and VEGF decreased in the GnRH + INS group compared with those in the INS group. This study showed that the administration of a single dose of GnRH delayed the negative effects of scrotal insulation on different seminal traits and revealed the pivotal role of GnRH in compensating testicular insulation in bucks.

## Introduction

Scrotal insulation is an acceptable model for examining the different harmful effects of local testicular hyperthermia in most domestic species, including bulls^1^ boars^2^, rams^3^, bucks^4^, and dogs^5^. However, substantial variations exist in the resulting thermal damage after insulation between species^5^, breeds^6^, and individuals^7^. In addition, testicular hyperthermia increases metabolism resulting in a need for more O2 and consequently hypoxia, which was considered one of the main causes of spermatogenic defects within the context of disrupted blood flow^8,9^. However, by altering testicular temperature and O2 content, testicular thermal damage primarily resulted from hyperthermia because heat-induced effects were neither replicated by hypoxia nor prevented by hyperoxia in rams^10^ and mice^11^.

Blockage in testicular vessels (e.g., varicocele and testicular torsion) or hypobaric hypoxia (e.g., reduced oxygen pressure at high altitudes) resulted in a compromised testicular blood flow, disrupting spermatogenesis, similar to changes after testicular hyperthermia^12,13^. However, increasing testicular blood flow in response to varying oxygen concentrations of inspired air could maintain the oxygen supply and avoid testicular hypoxia in rams^14^. Moreover, Herwig et al.^15^ have reported that sperm quality and quantity depend on blood perfusion within the testis. Doppler ultrasonography has been used as an alternative tool for evaluating reproductive parameters and testicular vascular dynamics in bulls^16,17^, rams^18^, and bucks^19,20^.

Furthermore, heat stress could increase cortisol secretion^21^ which in turn disrupted gonadotropin-releasing hormone (GnRH) pulsatility and negatively affected the reproductive process in ewes^22^. The peak of the GnRH-induced follicle-stimulating hormone (FSH) and luteinizing hormone surge significantly decreased in heat-stressed cows with low plasma estradiol^23^. In males, exogenous administration of a GnRH analog could affect testicular homeostasis by several mechanisms. For instance, it increased the expression of angiogenic factors^24^, inhibited thromboxane A2 (TXA2) receptors in testicular arteries^25^, decreased the resistance index of intratesticular vessels, and consequently increased the testicular blood flow^26,27^. Additionally, a single injection of the GnRH analog could improve the testicular blood flow in bucks^28^, and pretreatment with a GnRH agonist could enhance spermatogenesis, to some extent, in rat testes subjected to local heating^29^. Recently, Giriboni et al*.*^30^ have revealed the positive impact of GnRH administration in increasing testosterone concentration and sperm quality in bucks during the non-breeding season. However, knowledge is scarce on the effects of GnRH on testicular blood flow during testicular heat stress. Hence, we supposed that vasodilatation and the increase in testicular blood flow induced by GnRH could help maintain the proper thermoregulatory mechanisms in the testes and compensate for the negative effects of heat insulation in treated bucks.

Scrotal hyperthermia could induce several changes in the testicular genes and proteins^31,32^, leading to apoptosis in spermatocytes^33^ by activating various caspases^34^. Caspase-3 is a potent apoptotic marker and overexpressed in cases of spermatogenic arrest^35^ and heat-stressed testis^36^. Vascular endothelial growth factor (VEGF) is used as an angiogenic marker for testicular hypoxia^37^. A negative correlation between VEGF levels and germ cell damage has been reported in mouse models of unilateral cryptorchidism, and the expression of VEGF has been identified in the cytoplasm of Leydig, Sertoli, and germ cells^38^, in addition to the vascular endothelium of blood vessels^39^. Moreover, VEGF can induce Leydig cell proliferation and testosterone production^40^ and regulates spermatogenesis and self-renewal of spermatocytes^41^.

Based on the above-mentioned studies, it can be hypothesized that heat stress could induce testicular blood flow impairment, histopathological changes and adversely affect the testicular function. To the best of our knowledge, the effect of GnRH on blood flow of insulated testes is unknown. Thus, the present study evaluated the impact of exogenous GnRH administration on scrotal-insulated bucks along with its effects on sperm production and testicular vascular dynamics. Moreover, the testicular histopathology, and gene expression of caspase-3 and VEGF for the insulated bucks (with and without GnRH injection) were identified.

## Results

In this study, the scrotal temperature recorded for insulated testes was 34.63 °C ± 0.15 °C compared with 32.15 °C ± 0.7 °C for non-insulated testes (CON). All seminal parameters were adversely affected in bucks subjected to scrotal insulation (INS and GnRH + INS) for 92 days except the scrotal circumference (Tables 1 and 2).

**Table 1:** Effect of treatment on seminal parameters from bucks subjected to scrotal insulation (INS and GnRH + INS) or not (CON) for 92 days of insulation.

Parameter	Groups		
	Control	Insulated	GnRH + insulated
Volume (ml)	0.89 ± 0.02^a^	0.74 ± 0.03^b^	0.75 ± 0.02^b^
PH	6.98 ± 0.01^b^	7.51 ± 0.08^a^	7.53 ± 0.08^a^
Progressive motility (%)	84.14 ± 0.42^a^	37.71 ± 6.94^b^	46.43 ± 6.75^b^
Plasma membrane integrity (%)	86.43 ± 0.28^a^	38.07 ± 7.13^b^	48.89 ± 6.92^b^
Concentration (× 10^7^/ml)	377.61 ± 5.29^a^	184.46 ± 31.79^b^	202.93 ± 29.23^b^
Abnormalities%	8.71 ± 0.35^b^	25.71 ± 5.31^a^	29.86 ± 4.71^a^
Scrotal circumference (cm)	19.55 ± 0.12	19.36 ± 0.24	19.21 ± 0.26

^a,b,c^Means with different small superscript letters in the same row are significantly different (*p* < 0.05). (Duncan test).

**Table 2:** Interactions between time and treatment groups on seminal parameters from bucks subjected to scrotal insulation (INS and GnRH + INS) or not (CON) for 92 days of insulation.

Parameters	Time (days)	Groups		
		Control	Insulated	GnRH + insulated
Volume (ml)	0	0.80 ± 0.03	0.80 ± 0.04	0.78 ± 0.03
	5th	0.95 ± 0.06	0.90 ± 0.04	0.85 ± 0.03
	8th	0.93 ± 0.08	0.85 ± 0.03	0.88 ± 0.05
	12th	0.88 ± 0.05	0.80 ± 0.06	0.80 ± 0.04
	15th	0.88 ± 0.05^a^	0.53 ± 0.03 ^b#^	0.60 ± 0.04 ^b#^
	24th	0.90 ± 0.04^a^	0.55 ± 0.03 ^b#^	0.60 ± 0.04 ^b#^
	92nd	0.88 ± 0.05^a^	0.75 ± 0.03 ^b^	0.78 ± 0.03 ^ab^
PH	0	6.98 ± 0.03	7.03 ± 0.09	7.03 ± 0.03
	5th	6.93 ± 0.05^b^	7.23 ± 0.03 ^a^	7.30 ± 0.04^a #^
	8th	7.03 ± 0.03^b^	7.85 ± 0.09 ^a #^	7.75 ± 0.03 ^a #^
	12th	7.00 ± 0.04^c^	7.85 ± 0.03^b #^	7.98 ± 0.03 ^a #^
	15th	6.98 ± 0.03^b^	7.70 ± 0.07^a #^	7.75 ± 0.03^a #^
	24th	6.98 ± 0.03^b^	7.93 ± 0.05^a #^	7.95 ± 0.03^a #^
	92nd	6.98 ± 0.03	6.98 ± 0.03	6.98 ± 0.03
Progressive motility (%)	0	83.25 ± 1.03	82.50 ± 1.44	81.75 ± 1.18
	5th	83.75 ± 1.25^a^	68.00 ± 1.22 ^c #^	80.00 ± 0.00 ^b^
	8th	85.00 ± 0.41^a^	32.50 ± 1.44 ^c #^	57.50 ± 3.23 ^b #^
	12th	84.50 ± 0.50^a^	0.00 ± 0.00 ^c #^	9.50 ± 0.50 ^b #^
	15th	85.00 ± 0.00^a^	–	12.50 ± 1.44 ^b #^
	24th	82.50 ± 1.44^a^	–	–
	92nd	85.00 ± 2.04	81.00 ± 0.71	83.75 ± 1.25

^a,b,c^Means with different small superscript letters in the same row are significantly different (*p* < 0.05) (Duncan test). ^#^Significant different as compared to zero time in the same group (Paired T- test).

### Semen evaluation

The interactions between time and treatment on the seminal parameters from bucks subjected to scrotal insulation presented in Tables 2 and 3.

**Table 3:** Interactions between time and treatment groups on seminal parameters from bucks subjected to scrotal insulation (INS and GnRH + INS) or not (CON) for 92 days of insulation.

Parameters	Time (days)	Groups		
		Control	Insulated	GnRH + insulated
Plasma membrane integrity (%)	0	85.25 ± 0.25	84.50 ± 0.65	84.25 ± 1.11
	5th	85.75 ± 0.48 ^a^	69.00 ± 0.71 ^c #^	83.50 ± 0.29 ^b^
	8th	86.75 ± 0.48 ^a^	29.50 ± 0.29 ^c #^	62.00 ± 0.71^b #^
	12th	86.75 ± 0.25 ^a^	0.00 ± 0.00 ^c #^	14.25 ± 0.75 ^b #^
	15th	87.00 ± 0.41 ^a^	–	11.50 ± 0.65 ^b #^
	24th	86.25 ± 0.75 ^a^	–	–
	92nd	87.50 ± 1.66	83.50 ± 0.65	86.75 ± 1.31
Concentration (× 10^7^/ml)	0	352.75 ± 3.04	363.75 ± 8.00	368.75 ± 6.88
	5th	380.00 ± 2.04	343.75 ± 21.35	340.00 ± 22.73
	8th	378.75 ± 6.25 ^a^	192.00 ± 27.71^b #^	202.00 ± 4.55^b #^
	12th	385.00 ± 2.04 ^a^	21.00 ± 0.58 ^c #^	78.75 ± 2.39 ^b #^
	15th	383.25 ± 3.50 ^a^	0.00 ± 0.00 ^c #^	47.50 ± 3.23 ^b #^
	24th	380.50 ± 5.42 ^a^	0.00 ± 0.00 ^b #^	0.00 ± 0.00 ^b #^
	92nd	383.00 ± 5.87	370.75 ± 4.05	383.50 ± 2.90
Abnormalities%	0	9.50 ± 0.65 ^a^	7.00 ± 0.41 ^b^	10.50 ± 0.65 ^a^
	5th	6.50 ± 0.65 ^c^	40.00 ± 0.82 ^a #^	20.00 ± 0.41^b #^
	8th	9.00 ± 0.41 ^c^	58.00 ± 0.91 ^a #^	50.50 ± 0.65 ^b #^
	12th	9.50 ± 0.65 ^c^	70.00 ± 0.82 ^a #^	59.75 ± 0.85 ^b #^
	15th	11.00 ± 0.41^b^	–	61.25 ± 0.95 ^a #^
	24th	6.50 ± 0.65	–	–
	92nd	9.00 ± 0.41 ^a^	5.00 ± 0.41^c #^	7.00 ± 0.41^b #^
Scrotal circumference (cm)	0	20.00 ± 0.41	20.50 ± 0.29	20.13 ± 0.13
	5th	19.75 ± 0.25	20.50 ± 0.20	20.38 ± 0.32
	8th	19.75 ± 0.25	20.25 ± 0.25	20.00 ± 0.41
	12th	19.50 ± 0.29 ^a^	18.25 ± 0.14 ^b #^	17.50 ± 0.29^b #^
	15th	19.25 ± 0.25 ^a^	17.50 ± 0.29 ^b #^	18.25 ± 0.48 ^b #^
	24th	19.00 ± 0.41	18.50 ± 0.29 ^#^	18.00 ± 0.41 ^#^
	92nd	19.63 ± 0.38	20.00 ± 0.46	20.25 ± 0.75

^a,b,c^Means with different small superscript letters in the same row are significantly different (*p* < 0.05) (Duncan test). ^#^Significant different as compared to zero time in the same group (Paired T- test).

Herein for the INS group, the seminal volume decreased on day 15 after insulation being 0.53 ± 0.03, compared to 0.88 ± 0.05 for control group. The pH value of the semen increased (*p* < 0.05) on day 8 after insulation. Sperm motility decreased (*p* < 0.05) in the insulated bucks to 68% at day 5 and 32.5% at the 8th day and then fully stopped on the 12th day after insulation (Table 2). On day 12 of insulation, a significant decrease in the testicular circumferences was observed in the INS groups compared with that in the CON group (18.25 vs. 19.5 cm, respectively). A decrease (*p* < 0.05) in plasma membrane integrity, and normality percentage on the 5th day was observed after insulation (Table 3). The most common defects after scrotal insulation were bent tails (secondary abnormalities). After scrotal insulation, the percentage of abnormal sperm cells increased by 40% on the 5th day and reached 70% on the 12th day (Table 3). Additionally, Sperm plasma membrane integrity abruptly deteriorated on the 8th day to 29.5% in the INS group compared with 86.75% in the CON group. The sperm concentration was adversely affected from the 8th day and reached azoospermia after 15 days of insulation. Seminal evaluation data are presented in Tables 2 and 3.

The GnRH + INS group showed a delayed response to the thermal stress after scrotal insulation. For instance, sperm motility and plasma membrane integrity percentages decreased on day 8 (57.5% and 62.0%, respectively) compared with those in the INS group on the same day (32.5% and 29.5%, respectively). Moreover, the volume decreased on day 15 being 0.60% in comparison with 0.53% and 0.88% for INS and control groups, respectively (Tables 2 and 3). Interestingly, azoospermia was detected in the GnRH + INS group approximately 1 week later compared with INS bucks (Table 3).

All seminal traits and scrotal circumferences returned to pre-insulation values 92 days after the onset of scrotal insulation. The libido of all bucks was normal throughout the experimental period; however, the GnRH + INS group showed more sexual desire.

### Testicular blood flow

The PI values increased (*p* < 0.05) in the INS group for the first 3 weeks after scrotal insulation and then started to decrease reaching pretreatment values 5 weeks after insulation. In addition, the PI values increased in the GnRH + INS group for the first 2 weeks after scrotal insulation and then started to decrease reaching pretreatment values 4 weeks after insulation. Meanwhile, in the CON group, no significant differences in the PI values between the different weeks of the experiment (Table 4). The RI values increased in both the INS and GnRH + INS groups for the first 2 weeks after scrotal insulation and then started to decrease reaching pretreatment values 4 weeks after insulation. No significant differences in the RI values in the CON group throughout the experiment (Table 4). Additionally, the VP significantly decreased in the INS group during the 1st, 2nd, and 3rd weeks after insulation (*p* < 0.001) and then started to increase reaching pretreatment values by the 5th week after insulation. Meanwhile, the VP significantly decreased in the GnRH + INS group after insulation during the 1st and 2nd weeks (*p* < 0.05) and then started to increase reaching pretreatment values by the 3rd week after insulation (Table 4). Hemodynamics data are shown in Tables 4 and 5.

**Table 4:** Interaction between the insulation (with and without GnRH injection) and time on PI, RI and VP in bucks.

Parameters	Time (hrs)	Groups		
		Control	Insulated	GnRH + insulated
PI	0	0.77 ± 0.09 ^a^	0.52 ± 0.05 ^b^	0.69 ± 0.03 ^ab^
	1st	0.72 ± 0.09 ^b^	0.82 ± 0.04^# ab^	0.94 ± 0.05 ^a #^
	2nd	0.87 ± 0.17	1.07 ± 0.09 ^#^	1.10 ± 0.09 ^#^
	5th	0.74 ± 0.04	0.81 ± 0.21^#^	0.68 ± 0.09
	12th	.93 ± 0.17 ^a^	0.56 ± 0.04^b^	.67 ± 0.05^b^
	92nd	.77 ± 0.03 ^a^	0.36 ± 0.04^c #^	0.57 ± 0.04^b^
RI	0	0.56 ± 0.03 ^a^	0.36 ± 0.03 ^b^	0.50 ± 0.01 ^a^
	1st	0.50 ± 0.04 ^b^	0.48 ± 0.02 ^b #^	0.59 ± 0.01 ^a #^
	2nd	0.57 ± 0.06	0.61 ± 0.04^#^	0.65 ± 0.03 ^#^
	5th	0.48 ± 0.06	0.42 ± 0.04	0.44 ± 0.04
	12th	0.54 ± 0.07^a^	0.39 ± 0.02 ^ab^	0.46 ± 0.03^a^
	92nd	0.53 ± 0.01^a^	0.30 ± 0.03^c^	0.40 ± 0.03^b^
VP	0	26.30 ± 0.71^b^	28.62 ± 0.73 ^b^	37.60 ± 0.51 ^a^
	1st	24.90 ± 1.11^b^	33.56 ± 1.41^a #^	38.46 ± 1.79 ^a^
	2nd	26.22 ± 1.97 ^b^	27.89 ± 2.35^ab^	36.52 ± 2.56 ^a^
	5th	26.72 ± 1.50	23.83 ± 2.47	25.73 ± 1.41^#^
	12th	31.51 ± 2.78 ^a^	25.17 ± 1.16^b #^	20.51 ± 1.87 ^b #^
	92nd	26.87 ± 5.64 ^b^	26.49 ± 1.75^b^	40.58 ± 1.27 ^a^

^a,b,c^Means with different small superscript letters in the same row are significantly different (*p* < 0.05) (Duncan test). ^#^Significant different as compared to zero time in the same group (Paired T- test).

**Table 5:** The effect of insulation (with and without GnRH injection) and time on PI and RI in bucks.

	Groups		
	Control	Insulated	GnRH + insulated
PI	0.80 ± 0.04	0.70 ± 0.04	0.77 ± 0.04
RI	0.53 ± 0.02^a^	0.44 ± 0.02^b^	0.51 ± 0.02^a^
VP	27.09 ± 1.09^b^	28.00 ± 0.80^b^	33.23 ± 1.66^a^

^a,b,c^Means with different small superscript letters in the same row are significantly different (*p* < 0.05) (Duncan test).

### Testosterone

Overall, the serum concentrations of the testosterone decreased in INS group (2.98 ± 0.20%) when compared to control and GnRH + INS groups (4.90 ± 0.05 and 5.62 ± 0.33, respectively) (Table 6). In the INS group, the serum concentration of testosterone decreased (*p* < 0.05) 24 h (1.63 ± 0.03 ng/mL) after scrotal insulation and then increased gradually and rebounded to the normal level (4.55 ± 0.05 ng/mL) at the end of the experiment (92nd day) (Table 7). The GnRH + INS group showed a monophasic response to GnRH (11.70 ± 0.04 ng/mL) 12 h after injection and then returned to the normal level (Table 7).

**Table 6:** the effect of insulation (with and without GnRH injection) and time on the serum concentrations of the testosterone in bucks.

	Groups		
	Control	Insulated	GnRH + insulated
Over all means	4.90 ± 0.05^b^	2.98 ± 0.20^c^	5.62 ± 0.33^a^

^a,b,c^Means with different small superscript letters in the same row are significantly different (*p* < 0.05) (Duncan test).

**Table 7:** Interaction between the insulation (with and without GnRH injection) and time on the serum concentrations of the testosterone in bucks.

Time (hrs)	Groups		
	Control	Insulated	GnRH + insulated
0	4.55 ± 0.03	4.45 ± 0.06	4.56 ± 0.06
6th	4.65 ± 0.06^b^	4.53 ± 0.04 ^b^	5.50 ± 0.04 ^a #^
12th	5.02 ± 0.03^b^	4.45 ± 0.13 ^c^	11.70 ± 0.04 ^a#^
24th	5.10 ± 0.02^b^	1.63 ± 0.03 ^c #^	5.63 ± 0.04 ^a#^
48th	5.36 ± 0.06^a^	1.80 ± 0.04 ^c #^	5.11 ± 0.01 ^b #^
96nd	4.83 ± 0.08^a^	1.89 ± 0.02 ^b #^	4.58 ± 0.22 ^a^
192th	4.86 ± 0.03^a^	2.19 ± 0.02 ^c #^	4.58 ± 0.13 ^b^
384 th	5.10 ± 0.02^a^	2.30 ± 0.05 ^b #^	5.10 ± 0.02 ^a #^
768th	5.16 ± 0.04^a^	2.00 ± 0.04 ^c #^	4.75 ± 0.07 ^b^
92nd day	4.33 ± 0.05	4.55 ± 0.05	4.68 ± 0.05

^a,b,c^Means with different small superscript letters in the same row are significantly different (*p* < 0.05) (Duncan test). ^#^Significant different as compared to zero time in the same group (Paired T- test).

### Histological analysis

#### Semi-thin sections of testicular samples

**Figure 1:**
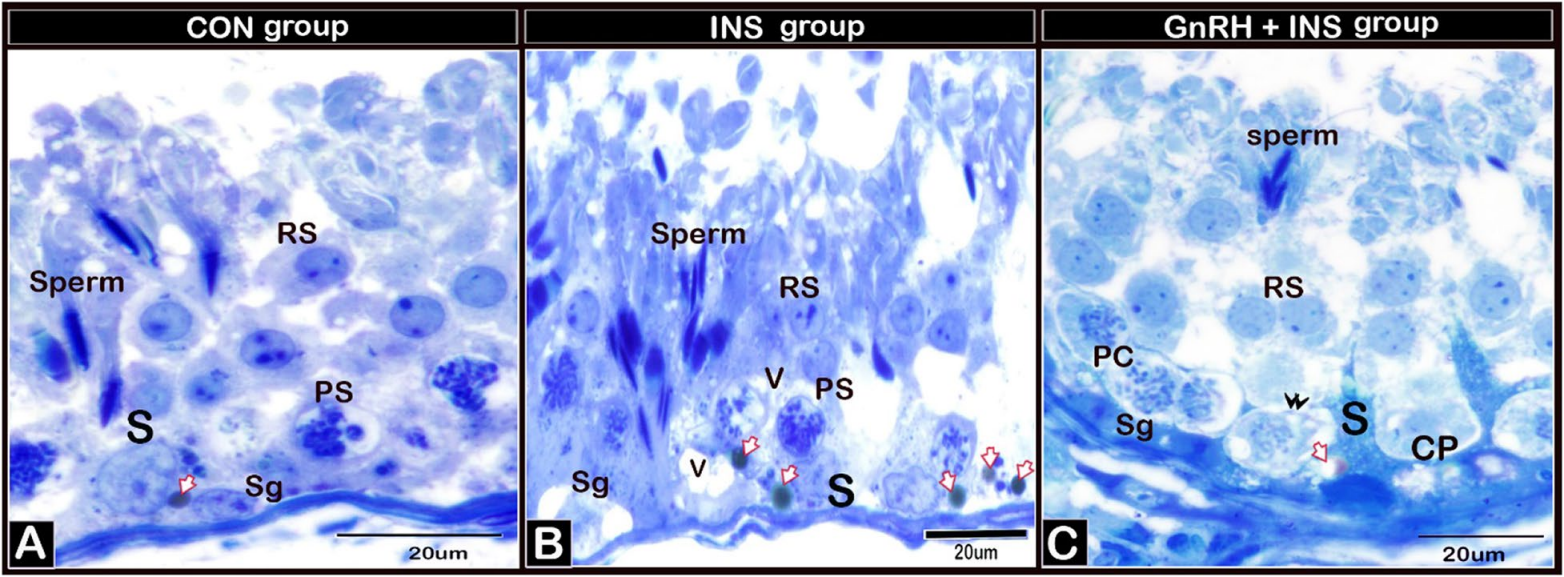
Photomicrographs of semi-thin sections of tubules from adult buck testes stained with toluidine blue (X1000 magnification). Testes in all groups exhibited a typical histological picture of functionally active mature seminiferous tubules. Sertoli cells showed differences in lipid inclusions and cell junctions in different experimental groups: A: Sertoli cells (S) in testis of CON showed low amount of lipid inclusion (arrow) and no vacuoles within the cytoplasm. B: INS showed marked accumulation of lipid droplets in Sertoli cells (arrow) and vacuolization (V) in both Sertoli and primary spermatocytes. C: GnRH + INS showed the Sertoli cells with scanty lipid inclusions, large cytoplasmic processes (CP) and no vacuoles in the Sertoli cells and primary spermatocytes. S: Sertoli cell, and Sg: spermatogonia, PS: primary spermatocytes, RS: round spermatid and sperm.

The testicular samples from all groups exhibited a typical histological picture of functionally active mature seminiferous tubules having regular spermatogenic development. The Sertoli cells of the bucks in the CON group had a low amount of lipid inclusions (Fig. 1A), whereas those in the INS group had large amounts of lipids (Fig. 1B). The cytoplasm of the Sertoli cells and primary spermatocytes was filled with vacuoles and did not maintain cellular contact with spermatocytes and spermatids in the INS group compared with those in the CON and GnRH + INS groups (Fig. 1B). For the GnRH + INS group, the Sertoli cells showed a larger cytoplasmic process with minimal lipid inclusions (Fig. 1C).

#### Immunohistochemistry expression of active caspase 3 and VEGF

**Figure 2:**
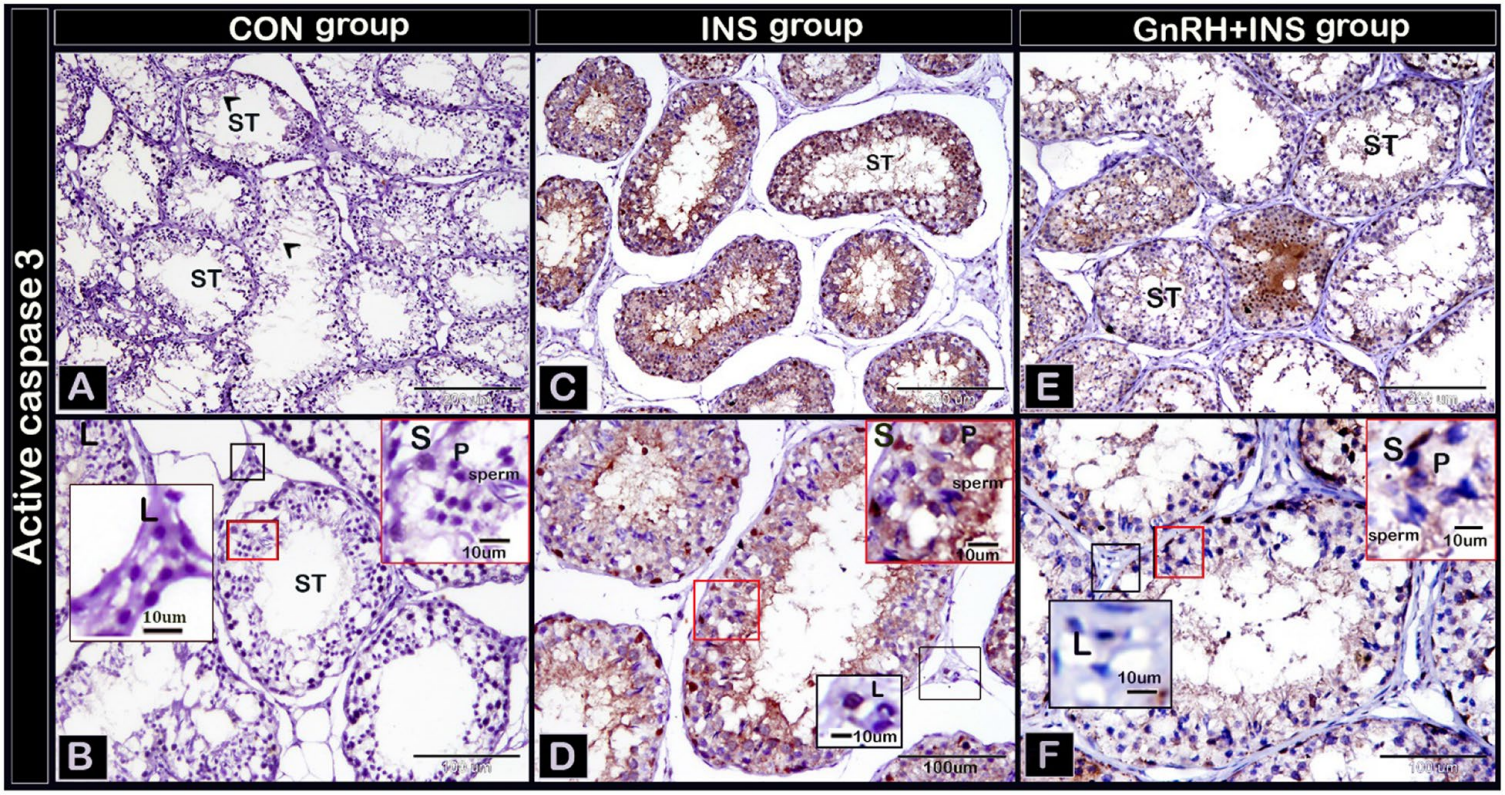
Photomicrographs of paraffin testicular sections stained with immunohistochemistry to detect the expression of active caspases-3: **A**, **C**, **E** (low magnification, X100), **B**, **D**, and **F** (higher magnification: X200). The CON group (**A** and **B**) showing a weak expression of caspases-3 immunostaining. Note: Arrowhead points to the reaction in sperm. The INS group (**C** and **D**) showing moderate expression of caspases-3 immunostaining. While, GnRH + INS group (**E** and **F**) showing the lowest expression of caspase-3 in Sertoli, germ and Leydig cells were decreased in comparison to insulated group. Abbreviation: ST: seminiferous tubules, S: Sertoli cells, P: primary spermatocytes, L: Leydig cells. The inserted squares highlight the reaction; red squares highlight the reaction in Sertoli and germ cells, and the black squares highlight the reaction in Leydig cells.

**Figure 3:**
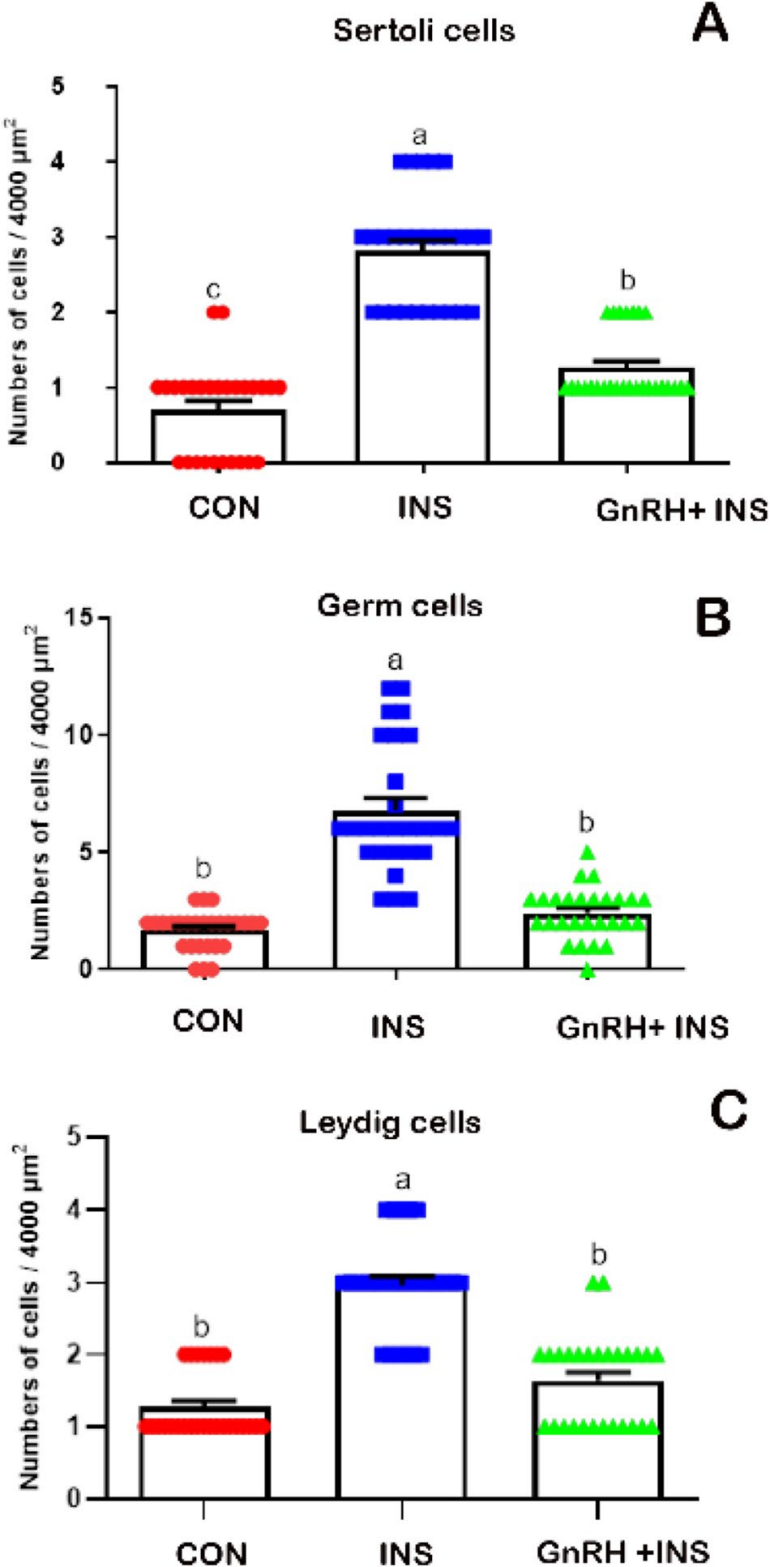
Numbers of apoptotic Sertoli (**A**), Germ (**B**) and Leydig (**C**) cells in seminiferous tubules of buck’s testis. Different letters indicate significant differences between the groups (*p* < 0.05). Results are presented as mean ± SEM.

The expression of active caspases-3 immunostaining was observed in Sertoli, germ, and Leydig cells. As expected, caspase-3 expression was weak in the CON group (Fig. 2A,B). The expression of caspase-3 in Sertoli, germ, and Leydig cells in the GnRH + INS group (Fig. 2E,F) was lower than that in the INS group (Fig. 2C,D). A negative image obtained using the CMEIAS color segmentation technique showed the reaction in different types of cells (Supplementary Fig. 1). Active caspase expression was evaluated by counting the number of caspase-immunoreactive cells in all experimental groups (Fig. 3). The highest number of active caspase-3 was observed in Sertoli (Fig. 3A), germ (Fig. 3B), and Leydig cells (Fig. 3C) in the INS group compared with that in the GnRH + INS group. The lowest number was detected in the CON group.

**Figure 4:**
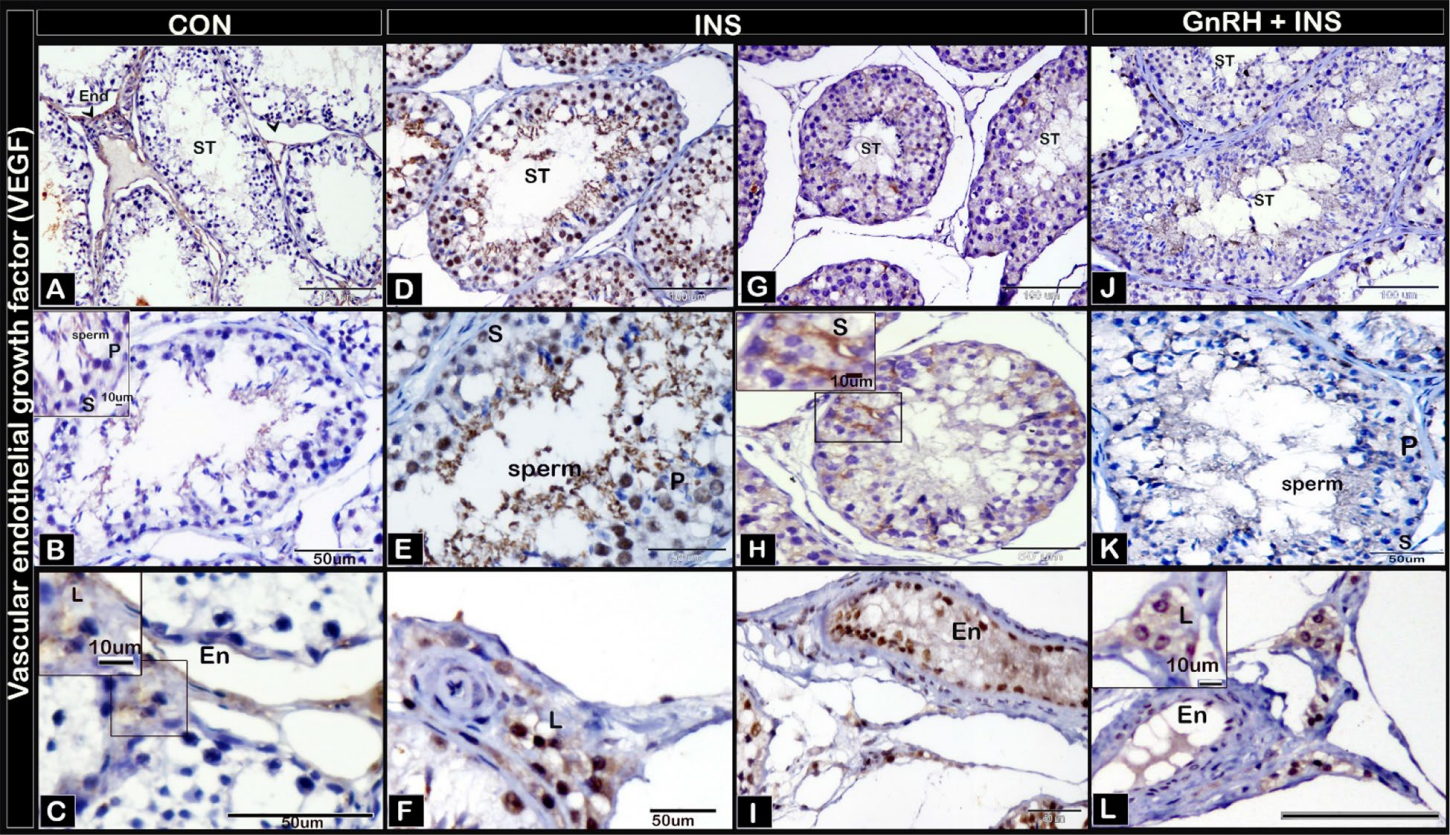
photomicrographs of testicular paraffin sections stained with immunohistochemistry to detect expression of vascular endothelial growth factor (VEGF): Control group (CON) (**A**, **B**, **C**) showed a strong expression for VEGF occurred in endothelium of blood vessels (En) and weak reaction in Sertoli (S), germ (primary spermatocyte, p) and Leydig cells (L). INS group (**D**, **E**, **F**, **G**, **H**, **I**) with a strong VEGF expression that distinguished in Sertoli cells and reacted in spermatogenic cells (Primary spermatocyte (p), Leydig cell (L), endothelium of blood vessels (En)). GnRH + INS (**J**, **K**, **L**) showed a weak VEGF expression which detected in Sertoli cells (S) and germ cell (primary spermatocytes, p), with a moderate reaction observed in Leydig cell (L) in comparison to INS group. Abbreviation ST: seminiferous tubules, S: Sertoli cells, En: endothelium of blood vessel, L: Leydig cells. The inserted squares showing the reaction B, H expressing the reaction in Sertoli and germ cells; C, L expressing the reaction in Leydig cells. Magnifications: (A, D, G, J, X200), (B_L, X400).

The expression of VEGF immunoreactivity staining was weak in Sertoli, germ, and Leydig cells and in the vascular endothelium of blood vessels (Fig. 4A–C) in the CON group. In the INS group, the reaction was relatively stronger in all cell types compared with that in other groups (Fig. 4D–I). The expression was weak in Sertoli and germ cells and moderate in Leydig cells in the GnRH + INS group compared with that in the INS group and nearly similar to that in the CON group (Fig. 4J–L). A negative image is represented as Supplementary Fig. 2.

**Figure 5:**
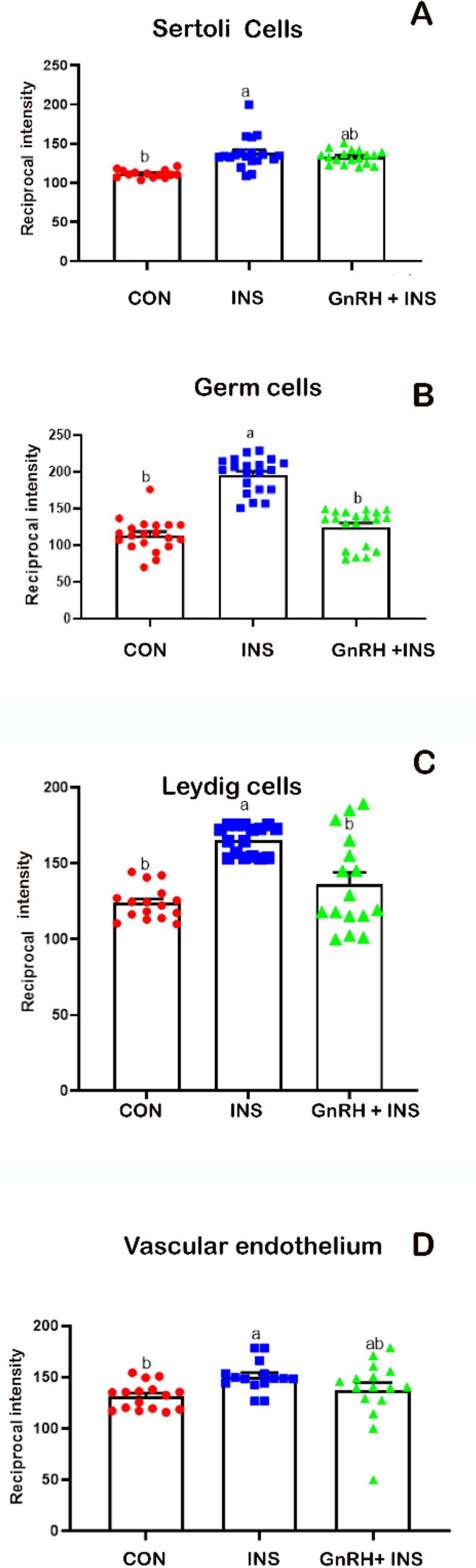
Reciprocal staining intensity of VEGF in (**A**) Sertoli, (**B**) germ, (**C**) Leydig cells and (**D**) vascular endothelium of buck’s testis. Different letters indicate significant differences between the groups (*p* < 0.05). Results are presented as mean ± SEM.

Regarding the reciprocal intensity of VEGF, the highest intensity of VEGF was observed in Sertoli (Fig. 5A), germ (Fig. 5B), and Leydig (Fig. 5C) cells and vascular endothelium (Fig. 5D) in the INS groups compared with that in the GnRH + INS group. The lowest intensity of VEGF was detected in the CON group. Supplementary Figs. 3 and 4 represent the negative and positive control immunostaining of active caspase-3 and VEGF, respectively.

## Discussion

In this study, the local heat stress generated by scrotal insulation decreased the scrotal circumference 12 days after the start of the experiment and returned to normal values 92 days after the onset of scrotal insulation. Such reduction in testicular volume can be explained by the destruction of germ cells^42^ and/or a reduction in testicular blood flow^43^ following thermal stress. Similar to our results, Cruz Júnior et al.^44^ have reported significant decreases in scrotal circumference and testicular volume 3 weeks after scrotal insulation in rams, which returned to normal values at the 5th week after insulation. As expected, the sperm parameters were negatively affected by scrotal insulation. For instance, sperm motility, morphology, plasma membrane integrity, and concentrations were adversely affected 5–8 days after insulation with the complete cessation of motility at day 12 in the INS group. These results conform to those reported by Santos and Simplício^4^, who have applied scrotal insulation for a week, and by Rahman et al.^45^, who have exposed bucks to hot weather. Additionally, Cruz Júnior et al.^44^ have reported that the declining values of sperm concentration and motility were observed 1 week after insulation in rams, reaching zero motility at week 3, and then increased to the pre-insulated values at week 10 or 11 depending on the breed. However, in rams, sperm motility decreased until the fifth week after 12 days of scrotal insulation without reaching azoospermia or the absence of sperm motility^13^. In bulls exposed to scrotal bags for 96 h, sperm motility and plasma membrane integrity were decreased and did not return to their normal values until the end of the experiment (28 days)^46^.

No changes in the sperm parameters occurred immediately after insulation because damaged spermatogenic cells do not enter the luminal compartment for spermiogenesis after heat stress. In bucks, spermatogenesis takes approximately 47 days^47^. Approximately 1 week was needed to show changes in the seminal parameters, and normality did not return until up to 11 weeks following the removal of the source of heat stress as shown in the current and previous studies^3,4,44,48^. It is noteworthy that the sperm parameters in the GnRH + INS group were less affected than those in the INS group throughout the experiment.

Likewise, testicular blood flow seems to change with testicular and/or environmental changes in temperature. In this study, both the RI and PI of the STA increased for the first 3 weeks after scrotal insulation and then returned to pre-insulation levels gradually from the 5th week. Moreover, the VP decreased in the GnRH + INS group for a shorter period and reached the pretreatment values earlier than that in the INS group. The results of this study confirm to those of a study on rats by Galil and Setchell^43^, who have found a reduction in the testicular blood flow in response to heat stress. In Sarda bucks, the mean RI values were lower in hot weather^19^. However, in bulls, the PI and RI did not show any significant changes after insulation until 120 h, but the VP was higher 10 min after insulation^49^. Notably, GnRH could counteract the effects of scrotal insulation on the testicular blood flow as its injection resulted in hastening the lowering of the blood flow and the resumption of testicular blood flow compared with those in the INS and CON groups. The exact mechanism by which GnRH increases the testicular blood flow is unknown. Samir et al.^20^ have reported induction of the testicular blood flow after human chorionic gonadotrophin or GnRH injection in intact bucks^28^ and rams according to Ungerfeld and Fila^50^. However, this study was the first to investigate the effects of GnRH injection on testicular blood flow during scrotal insulation.

The serum testosterone levels of the insulated bucks decreased compared with those of the controls. Accordingly, rams subjected to scrotal insulation for 72 h showed a lower serum testosterone concentration than non-insulated animals^51^. The low testosterone secretion from heated testes may be related to the decreasing in testicular blood flow that was shown in this study and previous investigations on rats^8^. Importantly, the testosterone concentrations increased in the GnRH + INS group 12 h after injection and did not reach subnormal values after insulation. The maximum testosterone response detected in this study (12 h) did not occur as early as those reported in 3–6-month-old bucks (2 h)^52^ and 10–12-month-old bucks (6 h)^53^. The difference in responses might be due to the effects of heat insulation on testosterone secretion, different routes of injection, age effects, different GnRH analogs injected, and breed differences.

Most importantly, in this study, GnRH reduced the effects of insulation on serum testosterone levels throughout the experiment. These results conform to those reported by Gábor et al.^54^, who have shown that serum testosterone levels increased after GnRH injection in bulls. Considering these investigations, the mechanism by which GnRH increases testosterone production may be due to its indirect effect on Leydig cells and their production of VEGF^40,55^ and/or its direct action on the testicular vasculature^53^.

In humans, the administration of GnRH could reduce the chemotherapy adverse effects on Sertoli cell functions during chemotherapy^56^. Morphological observations indicated that chronic hypoxia leads to degeneration and detachment of germ cells, folding of the basement membrane, and changes in lipid droplets in Sertoli cells^57,58^. These changes in droplet of Sertoli cells were similar to our observation after scrotal insulation. The results of this study elucidated the advantageous effects of GnRH in heat-stressed testes by reducing lipid inclusions in Sertoli cells. The direct mechanism by which GnRH protects Sertoli cells from lipid accumulations needs further investigation.

This study showed a marked dissociation of cellular contact between Sertoli and spermatogenic cells in the INS group compared with those in other groups. Similarly, Kanter, et al.^59^ have demonstrated enlarged intercellular spaces in both Sertoli and spermatid cells after scrotal hyperthermia in rats. Hence, the marked dissociation of the cellular contact between Sertoli and spermatogenic cells may lead to the destruction of the blood–testis barrier and the exposure of germ cells to the immunological attack and subsequently increase apoptosis of germ cells in the INS group. This explanation clarifies the lower number of apoptotic cells in the GnRH + INS group than that in the INS group. Accordingly, GnRH could stimulate the secretion of FSH and testosterone, which are essential in maintaining spermatogenic homeostasis by inhibiting death signals of germ cells^60^.

In this study, the relatively higher expression of caspase-3 in Sertoli cells of insulated bucks than in germ cells could support the typical caspase-dependent pathway for apoptosis and elimination of the destructed germ cells after thermal stress under the control of Sertoli cells^61^. In this study, scrotal insulation could decrease testicular blood flow, which led to hypoxic changes in the testis^9,62^ and subsequently increased the expression of VEGF. Hypoxia results in cell cycle arrest and apoptosis^59,63^. The findings of this study showed an increase in the expression of both caspase-3 and VEGF in Leydig cells in heat-stressed testes. These could explain the reduction of the testosterone level in insulated bucks in this experiment. However, the GnRH + INS group showed a higher blood flow and, subsequently, represented lower expressions of caspase-3 and VEGF than the INS group.

More importantly, the local heat stress in this study could be considered an acute testicular stress, which apparently recovered at the endpoint of this experiment. A single dose of GnRH analog could suppress apoptosis and support the angiogenic marker (VEGF) in heat-stressed testes that could explain the resulting long-standing adaptation of GnRH-insulated bucks at the level of testosterone production and testicular blood flow. The precise mechanisms by which GnRH counteracts the local heat stress of the testis deserve further research.

The results of this study show that the administration of a single dose of GnRH delayed the negative effects of scrotal insulation on different seminal traits and could antagonize its effect on testicular blood flow and testosterone production. These beneficial effects could increase male fertility, especially in cases of thermal stress through its positive influence on testicular blood flow and consequently spermatogenesis.

## Material and methods

### Ethics statement

The animal experiments described in this study were conducted according to the ethical guidelines and regulations established by the Animal Care Committee of Assiut University, Egypt. The study protocol was approved by the Committee for Ethics in Animal Experimentation of Assiut University (permit number: 17300319). The study was carried out in compliance with the Animals in Research: Reporting In Vivo Experiments (ARRIVE) guidelines^64^.

### Animals and study location

Twelve adult Egyptian male goats belonging to the Saidi breed (age, 18–22 months; average body weight, 22.75 ± 2.05 kg) were kept in a pen. All animals were offered wheat straw ad libitum and were fed a concentrate mixture containing 140 g of crude protein per kg diet twice a day (approximately 700 g concentrates/animal/day). Freshwater and mineral mixture blocks were freely available day and night. All bucks were healthy and in a good physical condition throughout the experiment. This study was conducted at the Veterinary Teaching Hospital, Faculty of Veterinary Medicine, Assiut University, Egypt, between July 2018 and November 2018.

### Scrotal insulation

Eight bucks were subjected to scrotal insulation for of cotton in between, with cords at the upper edge of the bag’s opening as previously described^65^. The period of insulation depended on the study by Hamilton et al.^13^ to evaluate the 12 days via the application of a bag consisted of a double layer of plastic and a layer lasting effects of local heat stress on sperm profile in rams. Four of the eight scrotal-insulated animals were treated previously with GnRH (GnRH + INS). The animals were intramuscularly injected (before the insulation) with 2-mL GnRH analog buserelin (Receptal-VET, Intervet, Unterschleißheim, Germany) containing 0.008-mg buserelin acetate. The remaining four insulated bucks that did not receive GnRH were considered the insulated group (INS). Control animals were not subjected to scrotal insulation or GnRH treatment (CON). To evaluate scrotal parameters (temperature and circumference) and color Doppler ultrasonography during scrotal insulation, the bags were removed and replaced after measurements. Scrotal temperatures were measured for three scrotal regions (proximal, middle, and distal) using an infrared thermometer (Model 22–325 infrared thermometer, RadioShack, USA). Scrotal surface temperature was recorded before every semen collection (7–8 AM). Scrotal temperatures, scrotal circumference, semen evaluations, and ultrasonographic parameters were assessed before the insulation (day 0), twice during scrotal insulation (day 8 and day 12), and weekly after bag removal until the seminal traits were equivalent to the pre-insulation values (day 92).

### Semen collection and evaluation

The bucks were isolated away from the does throughout the experiment. Semen samples were collected from all bucks early morning (7–8 AM) by using an artificial vagina and in the presence of a doe. After ejaculation, the semen samples were placed in a water bath at 37 °C to evaluate the seminal traits. Ejaculation volume was assessed directly from a graduated collection tube, and the pH of the semen samples was measured using pH-indicator strips immediately after the semen collection. Progressive sperm motility was evaluated by depositing a drop of semen diluted (1:10) in pre-warmed egg yolk citrate on a warm cover slide and cover slip^66^. Motility was evaluated as the proportion of forward-moving sperm cells under an optical microscope with a 40 × magnification by two experienced veterinarians. Plasma membrane integrity was assessed using the Vital Test (Halotech DNA SL, Madrid, Spain) as described by Dorado et al.^67^. Sperm morphology was examined using light microscopy evaluation on smears stained with Diff-Quick (Medion Diagnostics AG, Düdingen, Switzerland)^6^. At least 200 sperm cells per slide were counted to determine the percentage of sperm with abnormal forms. Abnormalities in sperm morphology were categorized according to the localization of the defects (head, midpiece, and tail), the place where the defect originated (primary: testes; secondary: epididymis; and tertiary: after ejaculation)^68^. To determine the concentration, a drop of semen diluted in water at the ratio of 1:9 (10-μL in 90-μL water) was placed in a Neubauer chamber, in which the sperm cells were counted.

### Testicular hemodynamic evaluation by Doppler ultrasonography

All Doppler ultrasonographic examinations were conducted using a duplex B-mode (grayscale) and color Doppler ultrasound instrument (ESAOTE Pie Medical MyLab One VET device, Firenze, Italy) equipped with a special veterinary (SV3513) linear array broadband transducer with frequencies ranging from 2.5 to 10 MHz. All ultrasonographic examinations were conducted by the same operator in the early morning (8–9 AM) and lasted 20–30 min for each buck at 0 h (before scrotal insulation), at different time points (1st, 2nd, 5th, and 12th weeks after insulation), and on the last day. The bucks were restrained without tranquilization or sedation. To eliminate the presence of air spaces, the hairs on both sides of the scrotum were thoroughly shaved, and the transducer was covered with a large amount of gel to improve ultrasonographic imaging.

In the bucks, the spermatic artery approaching the testis convolutes to form a convoluted region known as the testicular artery. The testicular veins collect to surround the artery to form the pampiniform plexus^69^.

Doppler analysis was conducted by identifying all vascular structures using grayscale ultrasonography and locating the largest longitudinal or oblique section of the testicular artery^69^. The angle between the Doppler beam and the long axis of the vessel was never more than 60° toward blood flow with the high-pass filter set at 50 Hz. To distinguish between a testicular artery and vein via Doppler analysis, an artery will have a typical waveform on the spectral graph corresponding to the arterial pulse in each cardiac cycle (systole and diastole), whereas the flow in the veins is almost constant without a pulse.

The size3h of the Doppler gate was adjusted during each examination to obtain a sequence of spectral Doppler graphs with symmetrical and distinct systolic and diastolic cardiac cycles. All color Doppler scans were performed at a constant gain, filter, and velocity range settings.

Regarding the Doppler parameters of the testicular artery, after the appearance of the spectral patterns of the testicular artery, testicular blood flow was assessed using the following parameters: pulsatility index (PI), resistance index (RI), and peak systolic velocity (VP). The formulas of the RI and PI are well established and have been reviewed (Ginther and Animal Reproduction: Color-Doppler Ultrasonography 2007). Three to five measurements were obtained for each parameter in different locations along the path of the supratesticular artery (STA). To minimize the variations in the recordings, the ultrasound settings (focus, gains, brightness, and contrast) were standardized, fixed, and used equally for all examinations. In this study, all calculations were performed automatically offline and stored on a flash memory device. A sequence of at least three successive symmetric blood flow waves was required to register the measurements during one cardiac cycle using an automatic trace. Following data collection, the Doppler images were transferred to a personal computer. The STA blood flow was determined using pulsed-wave Doppler ultrasonography. Doppler ultrasonography of the STA demonstrated a spectral graph with a wave-like pattern^70,71^.

### Blood collection and serum testosterone concentration evaluation

Blood samples were collected using jugular venipuncture into sterile plain collecting tubes. All blood samples were collected at the same time of the day throughout the experiment, in the early morning (before 8 AM), and then centrifuged at 2000 × g for 15 min. The sera were aspirated using Pasteur pipettes and stored at − 20 °C until hormone analysis. The samples were collected at 0 h before scrotal insulation at different time points (6, 12, 24, 48, 96, 192, 384, 768, and 1,536 h) and on the last day of the experiment (day 92). The serum levels of testosterone were measured as described by Kandiel and El Khawagah^72^ using an enzyme immunoassay test kit (Cat. No. BC-1115, BioCheck Inc., San Francisco, CA, USA). In brief, 10-μL serum, 100-μL testosterone–horseradish peroxidase (HRP) conjugate reagent, and 50-μL rabbit anti-testosterone reagent were thoroughly mixed (30 s) and incubated at 37 °C for 90 min. The micro-wells were washed, and 100-μL tetramethylbenzidine reagent was added to each well before incubation at room temperature for 20 min. The reaction was stopped, and the absorbance was assessed at 450 nm within 15 min.

The assay was based on the criteria provided by the manufacturer’s instructions, including the handling and storage of the samples and kits and assay procedures. Quality control specimens and different concentrations of standard were used with each run to verify assay performance. The sensitivity of the testosterone assay was 0.05 ng/mL. The intra- and inter-assay coefficients of variation for testosterone were 6.4% and 8.4%, respectively. Cross-reactions of various steroids were testosterone (100%), dihydrotestosterone (0.86%), androstenedione (0.89%), progesterone and cortisol (< 0.0001%), androsterone (1.0%), 17β estradiol (0.05%), and progesterone, epitestosterone, 17-OH-progesterone, estriol, cortisol, and dehydroepiandrosterone sulfate (< 0.05%).

### Histological analysis

#### Sampling and fixation

The testes were collected from all animals after slaughtering on the last day of the experiment (day 92). Histopathological investigations were applied 80 days after removal of the scrotal bag to ensure that testicular germ cells went through at least one spermatic cycle, minimizing the influence from the testicular insulation. The left testis and adjoining spermatic cord were obtained at the level of the spermatic cord and were slowly perfused through the testicular artery using a small amount of neutral buffered formalin to avoid the expansion of the testicular vessels.

#### Paraffin-embedded blocks preparation

Small blocks (1 × 1 × ½ cm) were taken from the testis and immersed in a special fixative (Wrobel–Moustafa fixative)^53^ for 24 h and were then processed as described by Yousef et al*.*^73^. Furthermore, 3–5-μm sections were cut using an RM 2125 microtome (Reichert Leica, Wetzlar, Germany) and mounted on glass slides. The sections were kept in an incubator at 40 °C for dryness and then stained with hematoxylin and eosin^74^.

#### Preparations of resin-embedded samples (Semi- thin sections)

Small pieces (2.0–3.0 mm) were fixed in Karnovsky fixative at 4 °C overnight^75^ and then were processed as described by Abdelhafeez et al*.*^76^. Semi-thin Sects. (1 µm) were cut using an ultra-microtome Ultracut E (Reichert Leica, Wetzlar, Germany) and stained with toluidine blue 33. A Leitz Dialux 100 microscope was used to examine the stained sections, and images were taken using a Canon digital camera (Canon PowerShot A95; Canon Inc., Tokyo, Japan).

#### Immunohistochemistry analysis

In this study, 5-µm paraffin-embedded sections were dewaxed, rehydrated, and rinsed in phosphate-buffered solution (PBS) (pH 7.4) three times for 5 min. Endogenous peroxidase was inhibited by adding drops of 3% hydrogen peroxide in methanol at room temperature for 20 min, followed by intense washing under running tap water for additional 10 min. For antigen retrieval, slides were placed in a 10-mL sodium citrate buffer (pH 6.0) and heated at 95–98 °C in a water bath for 20 min, followed by cooling for 20 min at room temperature. The sections were then rinsed in PBS (pH 7.4) three times for 5 min and covered by adding drops of blocking serum (Dako, Agilent Technologies Inc., Santa Clara, USA) for 5 min at room temperature to block nonspecific background staining.

Then, the sections were incubated with primary antibodies (anti-rabbit against the active caspase-3, Abacam, polyclonal, Clone: ab4051, at dilution 1:400 in BPS and anti-rabbit against the VEGF, Abacam, polyclonal, Clone ab53465, at dilution 1:100 in BPS). The incubation processes were performed overnight at 4 °C in a humid chamber. After incubation, the slides were washed with PBS (pH 7.4) three times for 5 min, followed by incubation for 30 min with an Envision + system HRP-labeled polymer anti-rabbit secondary antibody (k4003, Dako, Agilent Technologies Inc., Santa Clara, USA) at room temperature. After that, the slides were rinsed in PBS (pH 7.4) three times for 5 min, followed by incubation for 5–10 min at room temperature with 3,3′-diaminobenzidine (DAB) + substrate-chromogen, which resulted in a brown-colored precipitate at the antigen site. The sections were counterstained with Harris hematoxylin for 30 s. The sections were dehydrated using ethanol alcohol 90% and 100% I, II, cleared in xylene and covered by dibutylphthalate polystyrene xylene. We used the slides from insulated testes after showing azoospermia for positive and negative controls. The positive controls were performed following the aforementioned steps, and the negative controls did not have the primary antibodies, which were added during slide processing. A Leitz Dialux 20 microscope was used for examining the immunohistochemical staining, and the images were taken using a Canon digital camera (Canon PowerShot A95; Canon Inc., Tokyo, Japan).

Quantification and analysis of immunoexpression of active caspase-3 and VEGF were performed as follows:

The quantification of immunostaining of the nucleus and/or cytoplasm of active caspase-3 was performed using Image J (http://fiji.sc/Fiji). For counting, three sections from different areas of each animal with a magnification of × 100 in each group were used. In each section, we counted five squares of area approximately 4,000 µm2. The quantification of the staining intensity of VEGF was performed using the reciprocal intensity method described by Nguyen et al.^77^.

For the Center of Microbial Ecology Image Analysis System (CMEIAS) color segmentation (for the supplementary images of immunohistochemistry), negative images were obtained using the CMEIAS color segmentation software, which processes color images by segmenting the object of interest in the foreground from the background^78^. This process was conducted using the following steps: open image file with the CMEIAS color segmentation software, then select “Process” from the menu items, and subsequently choose “Negative image”.

### Statistical analysis

Semen parameter, testosterone concentration, PI, RI and VP were statistically analyzed using SPSS statistics 21 for windows (IBM SPSS, 2011). One-way ANOVA test and Duncan’s multiple range tests (Inc. 2004) were used to test the significance among groups and Paired-Sample T-Test was used to test the significance between different periods of measurement as compared to zero time in the same group. The data collected from immunohistochemistry investigations were statistically analyzed using one-way ANOVA via Graph Pad Prism version 5.0 (GraphPad Software, San Diego, CA, USA).

## Supplementary Information

Supplementary Information.
