# Supplementary material for: Exogenous gonadotropin-releasing hormone counteracts the adverse effect of scrotal insulation on testicular functions in bucks
Source: Sci Rep. 2022 May 12;12:7869. doi: 10.1038/s41598-022-11884-4 (PMC9098548; doi:10.1038/s41598-022-11884-4)

**Exogenous gonadotropin-releasing hormone counteracts the adverse effect of scrotal insulation on testicular functions in bucks**

Mohamed S. Yousef^1,*^, Gaber A. Megahed^1,^ Gamal F. Abozed^2^, Mohamed Hayder^2^, Hanan H. Abd-Elhafeez^3^, Mohamed S. Rawy^4^

^1^Department of Theriogenology, Faculty of Veterinary Medicine, Assiut University, Assiut, Egypt

^2^Animal Production Research Institute, Cairo, Egypt

^3^Department of Anatomy, Embryology and Histology, Faculty of Veterinary Medicine, Assiut University, Assiut, Egypt

^4^Department of Theriogenology, Faculty of Veterinary Medicine, Minia University, Minia, Egypt

^*^Corresponding author:

Dr. Mohamed S. Yousef, Ph.D.**,** email: [elmaamly20102002@yahoo.com](mailto:elmaamly20102002@yahoo.com), tel.: 002/01092294001

**Supplementary figure legends**

**Supplementary Fig 1.** Negative image of Photomicrographs of paraffin testicular sections stained with immunohistochemistry to detect the expression of active caspases-3: A, C, E (low magnification, X100); B, D, and F (higher magnification: X200). The CON group (A and B) showed a weak expression of caspases-3 immunostaining. Note: Arrowhead points to the reaction in sperm. The INS group (C and D) showed a moderate expression of caspases-3. While, GnRH+INS group (E and F) presented the lowest expression of caspase-3 in Sertoli, germ and Leydig cells in comparison to INS group. Abbreviation: ST: seminiferous tubules, S: Sertoli cells, P: primary spermatocytes, L: Leydig cells. The inserted squares showing the reaction; red squares showing the reaction in Sertoli and germ cells, while the black squares showing the reaction in Leydig cells.

**Supplementary Fig 2.** Negative image of photomicrographs of testicular paraffin sections stained with immunohistochemistry to detect expression of vascular endothelial growth factor (VEGF): Control group (CON) (A, B, C) showing strong expression for VEGF occurred in endothelium of blood vessels (En) and weak reaction in Sertoli (S), germ (primary spermatocyte, p) and Leydig cells (L). INS group (D, E, F, G, H, I) with a strong VEGF expression which distinguished in Sertoli cells and reacted in spermatogenic cells (Primary spermatocyte (p), Leydig cell (L) and endothelium of blood vessels (En)). GnRH+INS (J, K, L) showed a weak VEGF expression which detected in Sertoli cells (S) and germ cell (primary spermatocytes, p), with moderate reaction in Leydig cell (L) in comparison to INS group. Abbreviation ST: seminiferous tubules, S: Sertoli cells, En: endothelium of blood vessel, L: Leydig cells. The inserted squares showing the reaction B, H expressing the reaction in Sertoli and germ cells; C, L expressing the reaction in Leydig cells. Magnifications: (A, D, G, J, X200), (B_L, X400).

**Supplementary Fig 3.** Positive control for testicular sections from insulated testes during azoospermia stained with immunohistochemistry to detect the expression of Active caspase-3 (A, B) and vascular endothelial growth factor (VEGF) (D, E). Negative control for active caspase 3 and VEGF immunolocalization (C, F) was included.

**Supplementary Fig 4.** Negative image of positive control for testicular sections from insulated testes during azoospermia stained with immunohistochemistry to detect the expression of Active caspase-3 (A, B) and vascular endothelial growth factor (VEGF) (D, E). C and F represented the negative control for active caspase-3 and VEGF immunolocalization.

**Supplementary Fig 1.**
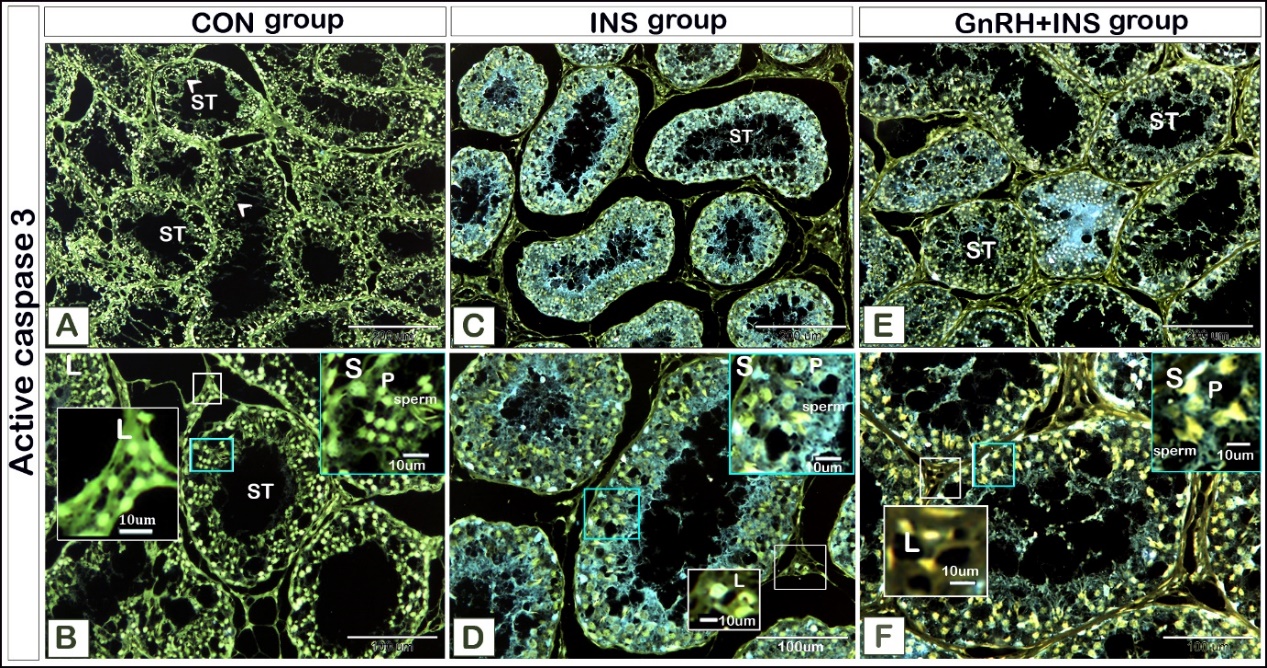


**Supplementary Fig 2.**


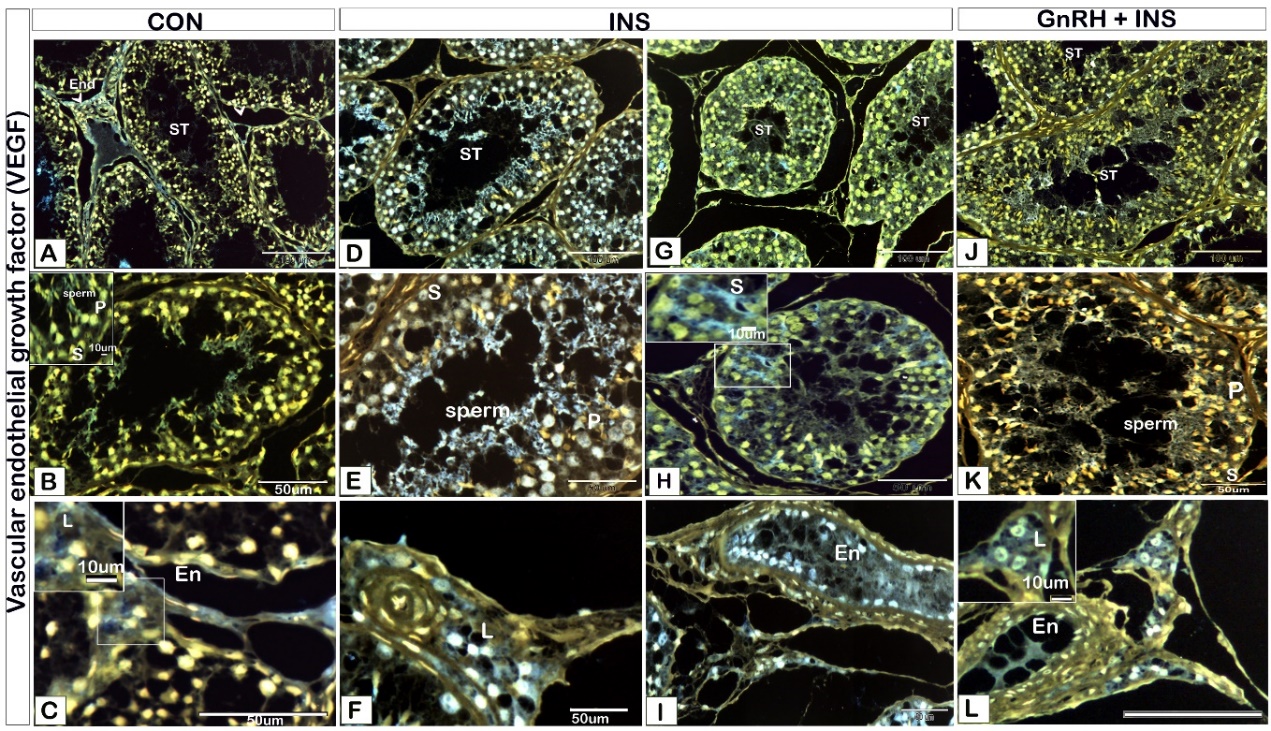


**Supplementary Fig 3.**


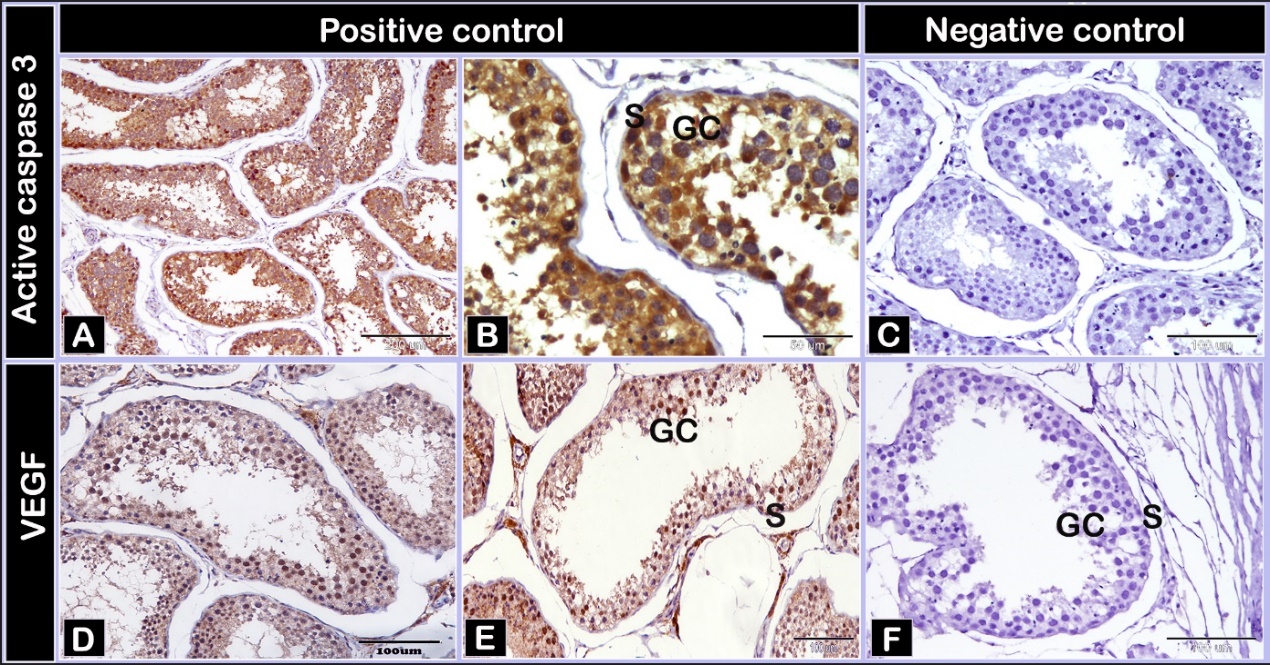


**Supplementary Fig 4.**


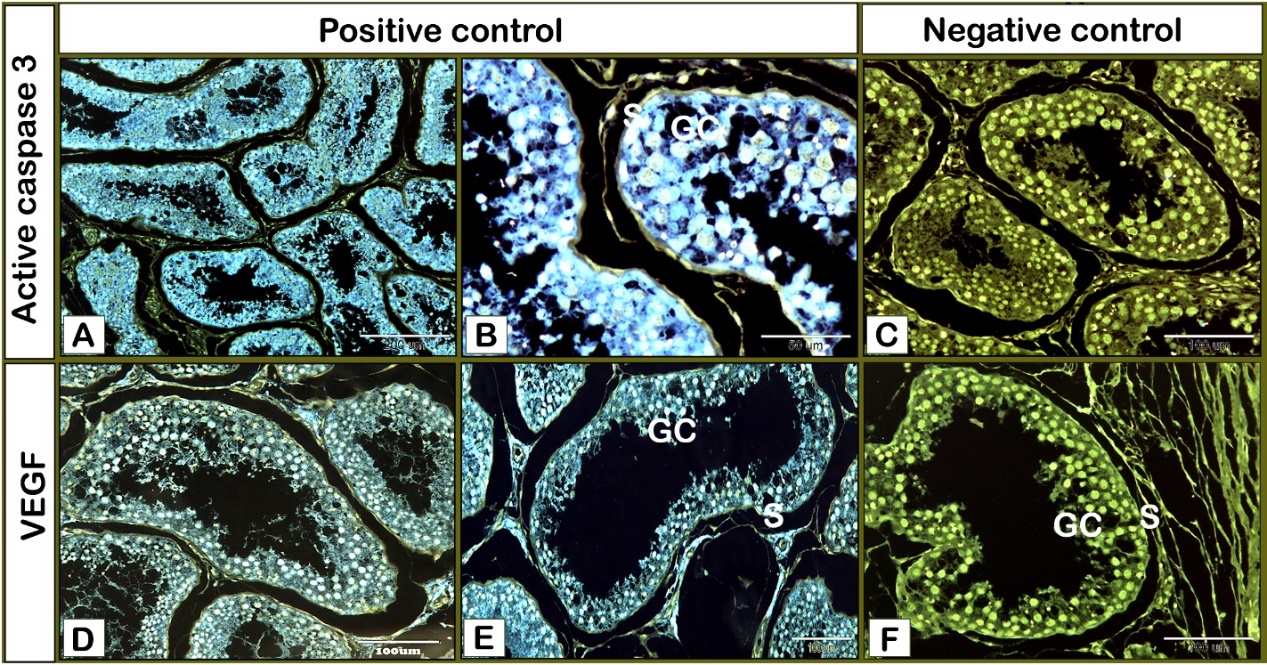

Supplement: Supplementary file 1 — Supplementary Information. [file 41598_2022_11884_MOESM1_ESM.docx]
